# Minimally Invasive Glaucoma Surgery: Safety of Individual Devices

**DOI:** 10.3390/jcm11226833

**Published:** 2022-11-18

**Authors:** Antonia C. Rowson, Daniel T. Hogarty, Dominic Maher, Lei Liu

**Affiliations:** 1 Alfred Ophthalmology Unit, Alfred Health, Melbourne, VIC 3004, Australia; 2 Royal Victorian Eye and Ear Hospital, University of Melbourne, Melbourne, VIC 3002, Australia

**Keywords:** ophthalmology, primary open-angle glaucoma, minimally invasive glaucoma surgery

## Abstract

Primary open-angle glaucoma progression in those already on maximal medical therapy has traditionally been treated with trabeculectomy, a surgical procedure that carries a high degree of morbidity. In the last few decades, significant advances have been made in the field of minimally invasive glaucoma surgery (MIGS) devices, which aim to defer or prevent trabeculectomy via less arduous surgical techniques in certain types of glaucoma. Although reviews have been published examining the efficacy of various MIGS techniques, no article synthesises the comparative safety of all available devices. We performed a literature review examining the safety of MIGS devices. Fifteen devices were included, variously attempting to increase aqueous outflow through the trabecular meshwork or the suprachoroidal space, shunting into the subconjunctival space, or reducing aqueous production through ciliary body ablation. Notably, the earliest product attempting to increase outflow to the suprachoroidal space, Alcon’s CyPass Micro-Stent, was withdrawn from the market due to concerns regarding increased corneal endothelial cell loss at five years post-implantation. All other devices were described as well-tolerated, with the most common adverse effects including hyphaema, intraocular pressure spikes, and device migration or obstruction. MIGS devices are purported to be uniformly safe, and many studies report no statistically significant increased complications beyond those associated with cataract surgery alone. It is important to note, however, the generally poor quality of current studies, with a dearth of randomised, or even prospective, data, and a large proportion of studies funded by device producers.

## 1. Introduction

Primary open-angle glaucoma represents the leading cause of irreversible blindness worldwide, and in an ageing world is projected to grow from 76 million patients in 2020 to 95.4 million in 2030 [1]. Although intraocular pressure (IOP) is not directly correlated to glaucoma severity, it has proven to be the only modifiable risk factor capable of reducing glaucoma progression and visual field loss [2].

Topical medication is considered to be the first-line treatment, with selective laser trabeculoplasty or more invasive surgical treatments second-line. Compliance with topical treatment is a known barrier to effective long-term IOP reduction; some reports suggest <25% of patients remain adherent to their prescribed medications after twelve months [3]. A procedure that can reliably decrease medication burden to one or, better, no medications is desirable given patient adherence is 44% when multiple medications are prescribed [4].

Trabeculectomy has long been a definitive surgical technique for lowering IOP; however, the rate of complications is significant. Intraoperative complications affect up to 10% of cases, and peri-operative complications between 50 and 57% [5,6,7]. Blebitis is an ongoing significant adverse effect with potentially serious consequences years after surgery, involving up to 5.7% of eyes [8,9]. Another conventional surgical technique, used with increasing frequency as a primary incisional glaucoma procedure, is glaucoma drainage device implantation. Otherwise known as tube shunt surgery, it is associated with a similar rate of complications to trabeculectomy; in Gedde et al.’s study, 20% of patients suffered early postoperative complications, growing to 22% at a later stage, and ultimately to 34% by three years postoperatively [10]. There were 2% of patients who required re-operation for vision-threatening complications: one instance each of conjunctival cyst formation, plate exposure, and tube retraction [10].

In recent decades, significant advances have been made in the development of surgical techniques that aim to tread the middle ground between these extremes; so-called minimally invasive glaucoma surgery (MIGS). A variety of devices now comprise the MIGS armamentarium, with general agreement that MIGS procedures involve the following components: (1) an ab interno corneal incisional approach, (2) minimal trauma, (3) a degree of IOP-lowering efficacy, (4) a high safety profile, and (5) rapid recovery [11]. The purpose of the following literature review is to better characterise the fourth of these claims, by quantifying the rate of complications associated with each available MIGS procedure.

## 2. Method of the Literature Search

**Figure 1:**
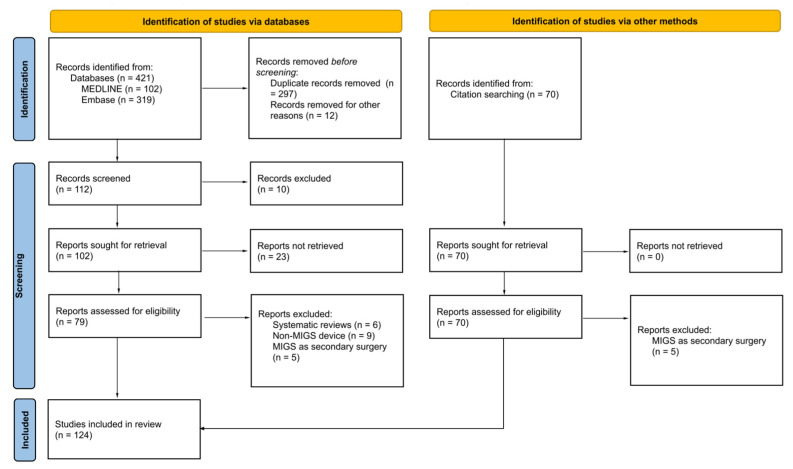
Method of the literature search.

A literature search was conducted in January 2022 independently by two authors (A.C.R. and D.T.H.) (see Figure 1). Two databases were used during the literature search: MEDLINE and Embase.

Search terms that were used are as follows:

1. “Minimally invasive surgical procedures” AND “glaucoma”;
2. “Minimally invasive glaucoma surgery”;
3. “MIGS”;
4. “Microinvasive glaucoma surgery”;
5. 1 OR 2 OR 3 OR 4;
6. “Complicat*”;
7. “Side effect*”;
8. “Adverse”;
9. 6 OR 7 OR 8;
10. 5 AND 9.

Full articles or abstracts that were written in English were included. Articles published in peer-reviewed journals within the last 20 years were selected for inclusion in this review if they were relevant to the aim of this article and advanced our understanding of complications following MIGS. Relevant publications found in returned articles’ references were also selected for inclusion. In the event of disagreement between authors as to the relevance of an article, we chose to include the disputed reference.

## 3. Results

### 3.1. Enhancing Aqueous Outflow through the Trabecular Meshwork

#### 3.1.1. iStent Trabecular Micro-Bypass Stent

**Figure 2:**
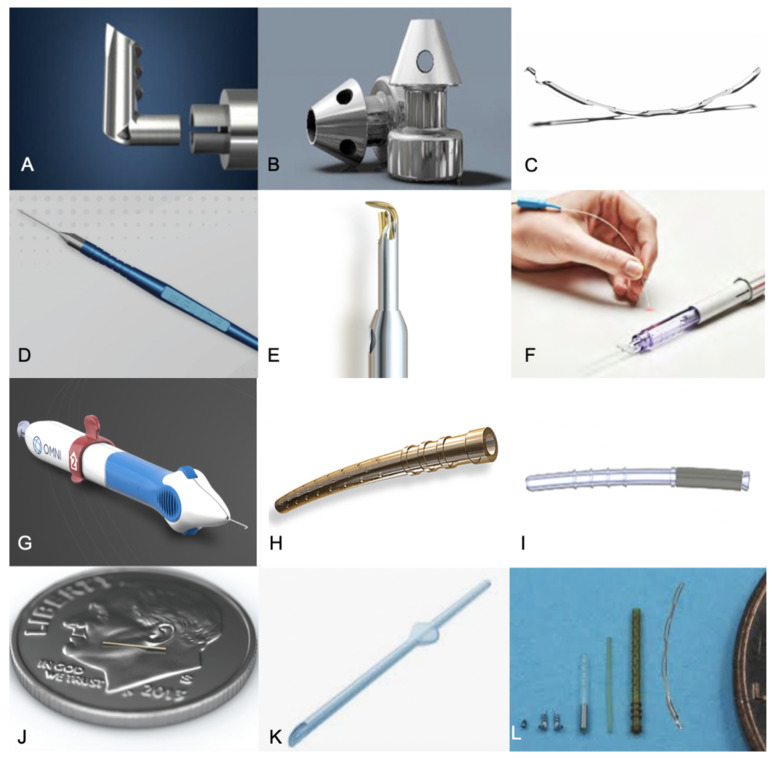
**(A**). iStent, (**B**). iStent Inject, (**C**). Hydrus Microstent, (**D**). Kahook Dual Blade goniotome, (**E**). Trabectome, (**F**). iTrack Microcatheter, (**G**). OMNI system, (**H**). CyPass Micro-Stent, (**I**). iStent Supra, (**J**). XEN Gel Stent, (**K**). PreserFlo MicroShunt, (**L**). Various stents arranged by size (images courtesy of Glaukos Corp, San Clemente, CA, USA.; Alcon Laboratories, Fort Worth, TX, USA; New World Medical Inc., Rancho Cucamongo, CA, USA; MicroSurgical Technology, Redmond, WA, USA; Ellex Inc., Minneapolis, MN, USA; Sight Sciences Inc., Menlo Park, CA, USA; Allergen Inc., Irvine, CA, USA; Santen Inc., Miami, FL, USA).

The iStent Trabecular Micro-Bypass Stent System (Glaukos Corporation, San Clemente, CA, USA) is a titanium, heparin-coated, L-shaped stent, 1.00 mm in length, that creates a channel for aqueous through the trabecular meshwork (TM) and into Schlemm’s canal [12] (see Figure 2).

Given its status as the first MIGS device to gain FDA approval (in 2012, with CE marking in 2004), multiple randomised controlled trials (RCTs) have been published that assess the function of the iStent. Ahmed et al.’s trial comparing outcomes for iStent and Hydrus implantation found that there were no significant differences in the rate of adverse events for either group across 12 months of follow-up; IOP spikes occurred in 5.2% of iStent participants [13]. Device obstructions occurred at a similar rate amongst groups, but cases of iStent obstruction were uniformly related to iris or other tissue blocking outflow (13.2% of participants) rather than secondary to peripheral anterior synechiae (PAS) formation [13]. The study also reported an unspecified amount of mild anterior chamber (AC) inflammation and corneal oedema in the first postoperative month, reportedly affecting a minority of patients [13].

Fea examined the differences between phacoemulsification alone and where combined with iStent insertion; his RCT of 33 patients had scant detail regarding safety outcomes, simply stating that there were no adverse events related to stent implantation [14]. Two stents were malpositioned, with one patient requiring further surgery to lower IOP [14]. Similarly, six stents were malpositioned in Fernandez-Barrientos’ study, with one stent extruding into the AC, apparently without associated inflammatory response [15]. In a population of 119 patients randomised to receive either one, two, or three iStents, no intraoperative complications such as hyphaema were reported, and adverse events postoperatively were limited to best-corrected visual acuity (VA) deterioration secondary to cataract progression [16]. There were no significant differences in safety outcomes between phacoemulsification alone and that combined with iStent insertion, in a study of 80 patients [17]. There were 6.25% of patients who developed microhyphaema postoperatively; these all resolved spontaneously within one week [17].

Stent obstruction affected 4% of cases in Samuelson et al.’s study of iStent and phacoemulsification versus phacoemulsification alone [18]. Where this occurred, 75% of obstructive events required stent repositioning surgically, with the remainder of cases amenable to nG:YAG laser [18]. Other complications were not significantly different between groups; notably, paracentesis was performed in 28% of combined treatment eyes and 27% of cataract-surgery-only eyes, for IOP spikes ≥ 10 mmHg above baseline. However, only 3% of eyes in each group required further medications or surgical intervention to reduce IOP thereafter [18].

The insertion of two iStents was compared to the effect of topical travoprost in Vold et al.’s study over three years [19]. One patient developed hyphaema and another iridodialysis intra-operatively, the cause of both was ascribed to patient movements [19]. No other operative or postoperative treatment-related adverse events were reported in either group [19].

Other studies of varying quality further corroborate the excellent safety profile of the iStent [13,20,21,22,23,24,25]; reported IOP spikes ranged from 0–22.5% of subjects, and hyphaema affected 0–9.7% of patients (see Table 1). The variable classification of IOP spikes does complicate direct comparisons. For instance, Ahmed et al. defined elevated IOP as >19 mmHg in their paper examining iStent outcomes [13], while Katz et al. used >18 mmHg [16], and Samuelson et al. >21 mmHg [18]. While some papers provided time-course-specific data on elevated IOP and associated interventions [16,19], others lack such details.

Several publications also examined the effect of iStent implantation on corneal endothelial cell loss (CECL) and found no significant difference compared to the effect of phacoemulsification alone at two years [25,26]. These data correspond with the results of a meta-analysis by Popovic et al., which found that 22.5% of iStent implantations were associated with some form of adverse event, most commonly IOP elevation or spike, stent blockage or malposition, and hyphaema [27] (see Appendix A; Table A1).

#### 3.1.2. iStent Inject

To further increase trans-trabecular outflow to Schlemm’s canal, a second-generation, smaller, and cone-shaped design (iStent inject^®^ Trabecular Micro-Bypass; Glaukos Corporation, San Clemente, CA, USA) and a modified injector preloaded with two iStent inject devices were developed [28]. In Fea et al.’s RCT comparing iStent inject with two ocular hypotensive medications, the IOP spike affected 1% of eyes, the same rate impacted by partial stent obstruction [28]. Another RCT of 505 eyes with mild to moderate primary open-angle glaucoma (POAG) found that a lower proportion of treatment group eyes (iStent inject + phacoemulsification) experienced a postoperative adverse event (at 54.1%) than those who underwent phacoemulsification alone (62.2%) [29]. While microhyphaema was noted in 3.9% of eyes, no eyes experienced hyphaema involving ≥10% of the AC, and while 6.2% of eyes showed signs of stent obstruction, only one in eight required laser intervention to treat this [29]. There was no statistically significant difference between rates of IOP spike or CECL in treatment versus control groups [29]. Seixas et al. found no rate of significant difference in adverse events between those undergoing cataract surgery with or without iStent inject implantation [30]. Other literature concurs that iStent inject implantation is a safe and well-tolerated procedure without sight-threatening sequelae [31,32,33,34,35,36]; where adverse events do arise, these are similar to those encountered with iStent insertion, namely IOP spikes, stent obstruction, and hyphaema, albeit at apparently lower rates [36].

#### 3.1.3. Hydrus Microstent

The Hydrus Microstent (Alcon Laboratories, Fort Worth, TX, USA) is an 8 mm long crescent-shaped open structure, curved to match the shape of Schlemm’s canal, and implanted using a preloaded hand-held injector. The microstent dilates Schlemm’s canal over three hours and bypasses the TM to provide direct aqueous access from the AC, without obstructing posterior wall collecting channels [37].

An RCT of 100 eyes examining cataract surgery with or without Hydrus stent insertion over two years found a significant increase in PAS formation in those with Hydrus Microstents (18.8% vs. 2.0%, *p* = 0.0077), most commonly within one clock hour of the device outlet. However, this was not considered clinically significant, as IOP and medication usage remained significantly less than the corresponding values of the cataract-surgery-only group [38].

The larger HORIZON RCT, examining 556 patients over three years, likewise found an increased rate of PAS amongst those with Hydrus stents (7.6%) relative to cataract surgery alone; once again, this was not associated with IOP elevation [39]. Interestingly, it found that the microstent induced an incremental non-statistically significant loss in mean endothelial cell count [39]; given the CyPass supraciliary stent was withdrawn after 5-year results found a newly significant CECL relative to phacoemulsification alone, the HORIZON group plans to assess specular microscopy annually to 5 years.

Hydrus efficacy over 12 months was compared to iStent insertion in the COMPARE RCT of 152 eyes; both were found to be well-tolerated with no significant differences in adverse events, none of which were sight-threatening [13]. The absence of serious safety concerns following Hydrus insertion has been further borne out by numerous lower-quality studies [40,41,42], with the most common adverse event being temporary postoperative IOP spikes that all settled conservatively.

#### 3.1.4. Kahook Dual Blade Goniotomy

The Kahook Dual Blade (KDB) (New World Medical Inc, Rancho Cucamongo, CA, USA) targets trabecular outflow by elevating and then excising a strip of TM tissue. The device, approved by the FDA in 2015, increases the direct flow from the AC into Schlemm’s canal without inserting a permanent implant into the eye. Two prospective randomised trials have examined KDB’s efficacy; Falkenberry et al.’s 12-month study of 164 eyes comparing KDB and iStent found a 31.7% rate of postoperative IOP spike, all successfully conservatively managed [43]. Furthermore, there was posterior capsule opacification (PCO) in 8.5%, and hyphaema persisting >1 week postoperatively in 3.7% [43]. Ventura-Abreu et al. conversely found no hyphaema in their 21 eyes undergoing KDB and cataract surgery, and did not specifically comment on IOP spikes postoperatively; the two postoperative adverse events noted in this study were isolated instances of corneal oedema and cystoid macular oedema, and reassuringly, corneal endothelial cell loss was non-significantly greater in the cataract-only group than the combined group at 12 months [44]. Other studies generally find hyphaema and IOP spikes to be the most common adverse events noted postoperatively; the former affects 4.5–34.9% of patients, and the latter 1.0–18.2% of patients [45,46,47,48,49,50,51,52]. Except in Tanito et al.’s study, where 5% of cases received AC washout [51], hyphaema was overwhelmingly conservatively managed. In fact, hyphaema may be anticipated as a sign of procedure success given that de-roofing Schlemm’s canal exposes collector channels to the AC. In a retrospective study of 116 eyes, Wakil et al. reassuringly found that where other complications such as corneal oedema, PCO, and cystoid macular oedema did occur, they were more common in combined KDB and cataract surgery rather than with KDB alone [52]. Tanito et al.’s 4% rate of macular oedema is consistent with the 4–11% rate post-cataract surgery [51,53], and 4.3% post-trabeculectomy [54].

#### 3.1.5. Trabectome

The Trabectome (MicroSurgical Technology, Redmond, WA, USA), FDA-approved in 2004, works by ablating a segment of TM and the inner wall of Schlemm’s canal [55]. Aside from a discontinued trial of 19 participants, no high-quality evidence in the form of RCTs has been published examining the precise effect of the Trabectome on open-angle glaucoma outcomes [56]. There is, however, a large base of less conclusive, non-randomised material.

In their retrospective analysis of 246 eyes with up to two years of follow-up, Ahuja et al. found 73% of patients had either micro- or macrohyphaema at day one, and that IOP spikes affected 22%; unusually, a further 5% of patients suffered delayed-onset hyphaema >2 months post-surgery [55]. Other studies reported rates of hyphaema between 4.72–100% and IOP spike between 2.06–28.9% [57,58,59,60,61,62]. While many other studies did not comment on the phenomenon, Esfandieri et al. noted a similar trend for delayed-onset hyphaema (>2 months postoperatively) to that observed in Ahuja’s work; 4.9% of Esfandieri’s cohort was affected [59]. Kono et al. reported one case of transient hypotony and one of endophthalmitis amongst 305 eyes studied; notably, PAS formation was 60.0%, a finding not often directly reported elsewhere [61].

Sato et al.’s prospective study of suture trabeculotomy in 64 eyes with follow-up through two years postoperatively found 50% of eyes had hyphaema on day one postoperatively, with two cases requiring AC washout secondary to IOP spikes [63]. Twenty-eight percent of eyes experienced IOP spike; all bar the two secondary to hyphaema were successfully managed with topical medication [63]. No cases of AC shallowing, wound leak, infection, or hypotony were noted [63].

#### 3.1.6. Gonioscopy-Assisted Transluminal Trabeculotomy

Grover was the first to describe the technique of gonioscopy-assisted transluminal trabeculotomy (GATT) in 2014; 30% of patients in this initial case series were noted to have a transient hyphaema. This may be anticipated, as the GATT technique involves circumferential deroofing of Schlemm’s canal using either a microcatheter (Ellex Inc., Minneapolis, MN, USA) or a suture via an ab inferno incision, resulting in reduced resistance to aqueous humour outflow [64]. Multiple subsequent studies have corroborated this one-week postoperative hyphaema rate of 30–40% [65,66,67,68]; conservative management results overwhelmingly in the resolution of this sign, with Rahmatnejad’s study reporting persisting hyphaema in only 6% of all subjects at one month [65]. IOP spikes were a poorly defined and subsequently widely varying phenomenon by study, affecting 0–18.1% of participants [66,69,70]. Rarely reported complications included transient microcystic corneal oedema [69], fibrinous uveitis [67], iridodialysis [71], and hypotony [66].

Sharkawi’s prospective case series of 103 eyes with pseudo-exfoliative glaucoma found an overall complication rate of 2.9%; one patient had hyphaema requiring AC washout, one patient had transient hypotony, and while 25 patients had some form of IOP spike, only one patient had hypertony persisting >2 weeks [72].

#### 3.1.7. TRAB360

The TRAB360 (Sight Sciences Inc, Menlo Park, CA, USA) device consists of a handpiece with a trabeculotome, a control wheel for advancing and retracting the trabeculotome, and a locking mechanism. The cannula is advanced to the iridocorneal angle, pierces the TM, and is advanced into and up to 180 degrees around Schlemm’s canal, then withdrawn to tear the external wall of the canal. By repeating the process in the opposite direction, it is possible to perform trabeculotomy encompassing 360 degrees [73]. In Sarkisian et al.’s retrospective study, 50.6% of patients experienced postoperative hyphaema, with a further 19.8% displaying signs of microhyphaema [73]. A further 6.2% experienced some degree of corneal oedema, with other complications more rarely seen; posterior vitreous detachment affected 2.5%, and there were single cases of iris trauma, neovascularisation, pain, IOP spike (secondary to steroid use), keratic precipitates, and retinal detachment [73].

#### 3.1.8. VISCO360

The VISCO360 system (Sight Sciences Inc, Menlo Park, CA, USA) is a non-implantable surgical device that delivers a predetermined amount of viscoelastic fluid to dilate up to 360° of Schlemm’s canal [74]. Tracer et al. found that, at 30 days postoperatively, hyphaema (1.7%) and IOP spikes (1.1%), and AC inflammation (<1%) were most common [74]. Up to 7% of eyes with baseline IOP > 18 mmHg experienced transient IOP elevation during follow-up, with paracentesis necessary only once [74]. Ondrejka et al. found a similar proportion of IOP elevation beyond one month (0.9%) [75]. The most common adverse event was the development of hyphaema, affecting 13% of eyes postoperatively, with all cases resolving with conservative management within seven days [75]. In a comparison between iStent insertion with cataract surgery and iStent + cataract surgery + VISCO360 canaloplasty, Heersink et al. found that all adverse events spontaneously resolved within three months of surgery; AC inflammation (6%), VA loss >2 lines (3%) and dry eye exacerbation (3%) were most common, with one case of hyphaema (1%) [76].

#### 3.1.9. OMNI System

The OMNI system essentially combines the TRAB360 and VISCO360 procedures, with a microcatheter inserted into Schlemm’s canal prior to a predetermined volume of viscoelastic fluid being injected to further dilate the canal and collector channels; Shlemm’s canal can then additionally be de-roofed to ameliorate juxtacanalicular outflow [77]. The device was FDA-approved at the end of 2017 [78]. Toneatto et al.’s study notes that intraoperative blood reflux into the AC was considered a sign of surgical success, and was noted in 100% of participants [77]; 2.5% of eyes developed persistent hyphaema requiring subsequent AC washout [77]. A 5.0% proportion of eyes showed transient hypotony of 4–5 mmHg; no AC shallowing or choroidal detachment was noted, and all cases were resolved with conservative management [77]. Conversely, 1.3% of eyes developed sustained IOP elevation beyond one month postoperatively [77]. The ROMEO study, retrospectively evaluating outcomes for 48 eyes, found 12.5% affected by mild AC inflammation and 6.3% by IOP elevation > 30 days postoperatively [78]. A 4.2% proportion of eyes developed hyphaema > 1 mm, and other adverse events including PCO (10.4%, in an exclusively pseudophakic population), cystoid macular oedema (6.3%), and corneal oedema (4.2%); details of the severity or requisite interventions for these complications were not reported [78]. Another arm of the same study, examining outcomes for 81 eyes following combined OMNI and phacoemulsification found lower rates of all aforementioned complications except PCO (17%) and corneal oedema (4.9%) [79].

#### 3.1.10. Ab Interno Canaloplasty

Ab Interno Canaloplasty (ABiC) (Ellex Inc., Minneapolis, MN, USA) uses a microcatheter inserted the length of Schlemm’s canal to break adhesions and stretch the trabecular plates as well as to withdraw herniated TM tissue from the collector channels. On removal of the catheter, the release of viscoelastic material ensures ongoing dilatation of the canal [80]. In accordance with its mechanism of action, intraoperative blood reflux has been noted in up to 100% of studied eyes [80]. In their study of 36 eyes, Davids et al. found that 2.8% developed postoperative hyphaema, with transient VA deterioration secondary to dispersed sanguis in 52.8% of patients; this finding had resolved conservatively in all cases by six weeks postoperatively [80]. No IOP spikes, hypotony, infection, wound leak, choroidal effusion, or haemorrhage was noted otherwise [80]. Hyphaema affected 20% of Kazerounian et al.’s study population, with one case of peripheral Descemet membrane detachment as the only other complication noted [81].

Gallardo retrospectively examined 24-month outcomes of ABiC using the iTrack canaloplasty microcatheter (Ellex Inc., Minneapolis, MN, USA); this features an atraumatic bulbous tip, designed to bypass collector channel ostia and push TM herniations out of the ostia with minimal tissue trauma, and with an illuminated fibre optic tip for added assurance of catheter location [82]. Scant information was given on the frequency and severity of complications, with conservatively managed microhyphaema as the only postoperative complication noted [82]. Likewise using the iTrack microcatheter to perform ABiC in conjunction with phacoemulsification, Gillmann et al. found rates of intraoperative complication to be 7.1%, comprising two cases of Descemet membrane detachment, one case of iris trauma, and one posterior capsule rupture [83]. Postoperatively, IOP spikes were overwhelmingly represented among adverse events, affecting 22.2% of the study population [83].

Al Habash et al.’s prospective case series detailing outcomes of combined GATT and ABiC in 20 eyes found IOP spikes (3 eyes, responsive to topical medication) and hyphaema (6 eyes, spontaneously clearing within one month) were the only complications noted [84].

#### 3.1.11. Summary

Available evidence suggests MIGS devices are well-tolerated, and those complications are able to be anticipated and generalised according to each device’s mechanism of action. iStent, iStent Inject, and Hydrus Microstent all bypass the trabecular meshwork by means of stent implantation; all share two of their five most common aggregate complications sampled from all included studies in this literature review, namely IOP spike, and device obstruction or stent occlusion. For certain individuals, differences in rates of complications may influence a clinician’s choice of device; for instance, IOP spikes are reported to affect between 1.8–22.2% of iStent patients [13,16,18,19,20,21,22,23,24,25], and, similarly, 1.06–18.6% of iStent Inject cases [28,29,30,31,32,34,35,36], but were only seen in between 1.9 and 6.45% of Hydrus implantations [13,38,39,40,41,42]. Notably, the number of Hydrus studies available for inclusion was smaller, and hence these data are possibly less reliable than for the two Glaukos devices, but in a patient with contra-indications to trabeculectomy or tube shunt surgery, this may nonetheless be a compelling consideration in Hydrus’ favour. Conversely, in a patient intolerant of mydriatic agents, the 0% rate of PAS formation following iStent and iStent Inject implantation may be valuable when compared against the 0–18.8% of Hydrus patients who developed this complication [13,16,18,19,20,21,22,23,24,25,28,29,30,31,32,34,35,36,38,39,40,41,42].

Four methods lower IOP by using tissue excision as a means of trabecular meshwork bypass: the KDB goniotomy, Trabectome, GATT, and TRAB360. All share hyphaema as their most common complication [43,44,45,46,47,48,49,50,51,52,55,57,58,59,60,61,62,63,64,65,66,67,68,70,71,72,73]; rates vary from between 0 and 34.9% with the Kahook device [43,44,45,46,47,48,49,50,51,52], to up to 95% of Trabectome cases [55,57,58,59,60,61,62,63]. It is worth noting that confidence intervals with this and all other complications are broad and will be more precisely established with the publication of further literature, but again these currently available data may inform a clinician’s device selection. Tissue excision methods may well be avoided in patients with sickle cell disease or other known bleeding diatheses, where the consequences of hyphaema may be more pronounced.

VISCO360, the OMNI system, and ABiC are all well-tolerated means of lowering IOP, based on available data. Principal complications for each device were variable, with only IOP spike noted consistently within the five most commonly reported complications for the group [74,75,76,79,80,81,83,84], which works to enhance aqueous outflow through Schlemm’s canal. Studies of VISCO360 reported only hyphaema and AC inflammation as affecting greater than 5% of participants [74,75,76]. The OMNI system conversely found PCO and AC inflammation to exceed the same thresholds [79], and ABiC to be most susceptible to postoperative IOP spikes and hyphaema [80,81,83,84]. Although IOP spikes may affect up to 22.2%, and hyphaema 20%, of ABiC candidates [80,81,83,84], the rates of these and other complications within this group are less than the principal complications associated with devices of alternate mechanisms of action.

### 3.2. Enhancing Aqueous Outflow through the Suprachoroidal Space

#### 3.2.1. CyPass Micro-Stent

The CyPass Micro-Stent (Alcon Laboratories, Fort Worth, TX, USA) is a 6.35 mm fenestrated polyimide stent designed to be inserted into the supraciliary space; this is the only MIGS device that targets the suprachoroidal space as a means of improving uveoscleral outflow. Early non-randomised studies found CyPass to be both efficacious and well-tolerated, with no significant safety concerns [85,86,87,88]. The COMPASS trial, the Alcon-funded RCT examining the device’s efficacy, likewise found no safety concerns up to the two-year mark [89]. However, the further three-year extension to this trial, entitled the COMPASS-XT study and assessing long-term safety, found a significantly lower corneal endothelial cell density in those with CyPass implants relative to control group members [90]. A more detailed examination of these data in a separate paper found endothelial cell density was reduced by 20.4% (95% CI 17.5–23.5%) in the CyPass and phacoemulsification group relative to 10.1% (95% CI 6.3–13.9%) in the phacoemulsification-only group [91]. In light of these results, CyPass was pulled from the market in 2018 [92] (see Appendix A; Table A2).

#### 3.2.2. iStent Supra

iStent Supra (Glaukos Corporation, San Clemente, CA, USA) is a 165 μm heparin-coated stent, inserted via a preloaded injector from the trabecular meshwork into the suprachoroidal space. The device is yet to be FDA approved, with results of a Glaukos-funded RCT examining the effect of stent insertion in combination with cataract surgery yet to be published [93]. A prospective study of 80 patients who underwent combined insertion of two iStents, one iStent Supra, and were prescribed topical prostaglandins postoperatively, found that this triple therapy was well-tolerated [94]. Bar one unsuccessful iStent Supra insertion, secondary to an obscured visual field post-iStent implantation, there were no intraoperative complications such as hyphaema [94]. Furthermore, only twelve individuals had a deterioration in BCVA ≥ 3 lines across four years of follow-up, and this was attributed to the progression of cataracts in eleven cases [94]. Measures of central corneal thickness, visual field mean deviation and pattern standard deviation, and cup-to-disc ratio remained stable for four years, with further follow-up to five years planned by investigators [94].

#### 3.2.3. Summary

Although affecting the comparison of all devices to some degree, the dearth of data published with regard to the iStent Supra device, in particular, renders comparison against its competitor, the CyPass Micro-Stent, difficult. The latter stent has of course been withdrawn from the market [92], and while available data from iStent Supra are yet to report outsized CECL, there is only one prospective study currently published detailing complication rates for this device. Both devices are associated with BCVA loss over time [85,87,88,89,90,94]; in the case of iStent Supra, 15% of patients noted deterioration of three or more lines of visual acuity on testing [94]. Absent any other reported complications, iStent Supra superficially appears to be a safe device, but manufacturer Glaukos has not made the device available to commercial markets, precluding its use (and further testing) in real-world settings.

### 3.3. Shunting Aqueous Outflow into the Subconjunctival Space

#### 3.3.1. XEN Gel Stent

No RCTs examining the efficacy of the XEN Gel Stent (Allergan Inc, Irvine, CA, USA), FDA-approved in 2016, have yet been published [95]. Offered in earlier formats as the XEN140 and XEN63 Gel Stents, with lumen diameters of 140 or 63 μm, respectively, the current model is the XEN45 Gel Stent, with a lumen of 45 μm diameter [96]. The stent is passed through the iridocorneal angle, bypassing the TM, and allowing aqueous drainage to the subconjunctival space [97]. Adjunctive antifibrotic agents are often applied to the conjunctiva as part of the procedure, given an adequately-functioning filtering bleb is necessary for the stent’s optimal success [97].

Postoperative hyphaema affected 3.8–5.6% of eyes [96,97,98], with IOP spikes affecting 2.6–21.5% [96,97,99,100], and notably stent migration necessitating removal in 0–2.3% [96,97,98,100,101,102]. Of note, mitomycin C or another antifibrotic agent were invariably utilised at the time of stent insertion in these studies, affecting the subsequent characteristics of the conjunctiva. Hypotony is another frequent postoperative finding, affecting 6.1–34.7% of eyes [97,99,100,101,102,103,104], with choroidal detachment, happily, rather less frequent (0–17%) [96,97,100,101,102,105].

Endophthalmitis was a complication in reports by Baser, Heidinger, Karimi, and Reitsamer, affecting 0.4–3% of participants in these studies [98,99,100,103]. Another complication secondary to the construction of a filtering bleb was the necessity for revision or needling, affecting 22.1–43% of eyes [97,98,99,100,101,102,103,105] (see Appendix A; Table A3).

#### 3.3.2. PreserFlo MicroShunt (Formerly InnFocus MicroShunt)

Yet to receive FDA approval, but licensed for use in European, Canadian, and Australian markets, the PreserFlo MicroShunt (Santen Inc., Miami, FL, USA) is a biocompatible stent inserted via an ab externo approach [106]. The device allows aqueous flow from the anterior chamber to a posterior bleb raised under the conjunctiva and Tenon’s capsule [106]. A one-year, prospective, randomised study of 527 patients comparing outcomes of PreserFlo insertion to trabeculectomy found that the former was associated with significantly fewer postoperative interventions (40.8% vs. 67.4%, *p* < 0.01) [107]. Furthermore, hypotony was also less likely in the PreserFlo intervention group (28.9% vs. 49.6%, *p* < 0.01), but rates of hypotony requiring intervention did not reach statistical significance between the two interventions [107]. Moreover, vision-threatening complications were roughly equivalent between groups, affecting 1.0% of PreserFlo implantations [107]. Other common complications included elevated IOP requiring treatment (25.3–32.9%), subconjunctival bleeding or hyphaema or microhyphaema (6.1–16.7%), bleb leak (6.6%), and choroidal effusion or detachment (4.6%) [107].

Rates of eyes that require bleb needling range from 5.0–19.3% [106,108,109,110,111,112,113], with the exception of a remarkable 62.5% rate in Ahmed et al.’s case series of eight individuals [114]. Other notable complications across the currently published literature include hypotony (1.7–39.0%), corneal oedema (0–17.4%), shallow/flat anterior chamber (2.5–13.0%), choroidal detachment (2.0–12.9%), hyphaema (2.5–20.0%), posterior synechiae (0–4.3%) and pupillary capture (0–4.3%) [106,108,109,110,111,112,113,115,116]. Beckers et al. found a statistically significant rate of difference in certain complications according to mitomycin C concentration used intra-operatively (0.02 vs. 0.04 mg/mL) [106]; further studies are needed to clarify the effect of this. Stent exposure has rarely been reported in the literature; Bunod et al. published a case report of two such instances, and there have been at least five other patients in similar circumstances [109,113,116,117,118]. XEN stent exposure is conversely estimated to occur in up to 2.3% of eyes [96,97,98,99,100,101,102,103,105].

#### 3.3.3. Summary

Devices that shunt aqueous outflow into the subconjunctival space, namely the XEN Gel Stent and the PreserFlo MicroShunt, require the creation of a conjunctival bleb in order to function effectively. This promotes a host of bleb-related complications that are not seen in other devices. For instance, bleb needling, hypotony, and choroidal detachments or effusions are all within the five most commonly reported complications for each of these devices [96,97,98,99,100,101,102,103,105,106,107,108,109,110,111,112,113,114,115,116]. For patients unwilling to undergo multiple procedures, the bleb needling requisite for 22.1–43.4% of XEN stents to function [96,97,98,99,100,101,102,103,105], and necessary up to an average of 1.5 times per PreserFlo shunt implanted [106,107,108,109,110,111,112,113,114,115,116], may be a significant contraindication against the use of these devices. The majority of research suggests lower rates of needling are required with PreserFlo than with XEN stents [96,97,98,99,100,101,102,103,105,106,107,108,109,110,111,112,113,114,115,116]; this may relate to the manner of device insertion. PreserFlo is usually inserted via an ab externo approach, and XEN is traditionally implanted through an ab interno incision.

It is important to recall that bleb-related complications can ultimately be sight-threatening. Stent exposure and endophthalmitis are two particularly concerning sequelae of subconjunctival device implantation and, respectively, occur in up to 2.3% and 3% of XEN insertions [96,97,98,99,100,101,102,103,105]. They are posited to be rarer in the case of PreserFlo [109,113,116,117,118].

### 3.4. Reducing Aqueous Production by Ciliary Body Ablation

#### 3.4.1. Endocyclophotocoagulation

Endocyclophotocoagulation (ECP) allows for direct visualisation of the ciliary processes and ablation of the pigmented ciliary epithelium, causing thermal damage to the non-pigmented ciliary epithelium, which reduces aqueous humour production [119].

There has been one randomised controlled trial to date examining the effects of combined ECP and cataract surgery versus cataract surgery alone, in the years following the technique’s development in 1992 [120,121]; the study population however comprises those with primary angle-closure glaucoma, which is not within the scope of this review.

Koduri et al. performed a large retrospective analysis of 4423 eyes undergoing phacoemulsification only (4242) versus phacoemulsification and ECP (181), to determine the risk of persistent anterior uveitis (PAU) [122]. It found a significant increase in the likelihood of PAU amongst those undergoing ECP; 1.7% of phacoemulsification-only eyes developed PAU, compared to 14.9% undergoing phaco/ECP (*p* < 0.0001) [122]. In stratified analysis, this risk was particularly pronounced for Caucasian patients, with an odds ratio of 17.9 (95% CI 7.8–41.1, *p* < 0.0001) for the development of PAU after phaco/ECP [122]. The time to resolution of AC inflammation and duration of topical steroids were not significantly different by procedure, where PAU did occur [122].

Izquierdo et al. compared outcomes following ECP with and without additional KDB goniotomy [119]. IOP spikes affected 11.11% of the former and 31.81% of the latter cohorts [119]. Corneal oedema affected 22.45% of eyes but resolved within the first postoperative week, and where hyphaema did occur, this remained <1 mm in height [119] (see Appendix A; Table A4).

#### 3.4.2. Summary

The sole means of reducing IOP via ciliary body ablation and subsequent reduction in aqueous production, ECP is apparently well-tolerated. Complications affecting ECP were found to affect all other MIGS to a greater or lesser degree; IOP spikes, corneal oedema, keratitis, PCO, anterior uveitis, and CMO [119,122]. However, adequate comparison and assessment are constrained by limited available data; only two studies were found that detailed ECP complications [119,122]. Again, this speaks to the need for further research, and more particularly high-quality prospective data, to provide further certainty of all possible complications associated with MIGS usage and a more precise estimation of their likelihood.

## 4. Conclusions

Although there are published reviews examining the efficacy and/or safety of various MIGS devices, we have performed the most comprehensive review to date of published safety outcomes for 15 currently and previously available MIGS devices. We categorised each device according to its mechanism of action and then summarised the most common adverse outcomes within these classes.

With the notable exception of the CyPass Micro-Stent, which was only withdrawn after studies of five-year efficacy uncovered an accelerated rate of CECL associated with the device, all other marketed MIGS devices have been found to be uniformly safe in all reported literature. In fact, a significant proportion of studies compare device implantation and phacoemulsification to phacoemulsification alone, and found no significant differences in the rate of complications between groups; the implication is therefore that MIGS device insertion is as safe as cataract surgery. The potential severity of bleb-related complications unique to devices that target the subconjunctival space (XEN and PreserFlo) are worth careful consideration. However, although outside of the purview of our review, we are obliged to acknowledge these are a particularly effective class of MIGS for IOP reduction.

Notably, many devices lack long-term data, given the recency of their development, and many studies comprise poor quality evidence, with retrospective case series overwhelmingly informing the body of evidence relative to more robust methodologies; the former can only include documented outcomes, which are likely to vary by clinician. Open-label reporting of outcomes, the inability to blind participants or researchers to assigned study groups, and small sample sizes are common deficiencies in the studies included above. Furthermore, a large proportion of the literature has been financed by companies that manufacture MIGS devices, which may provoke reporting bias. Further research with high-quality design and independent funding would improve the confidence with which clinicians are able to recommend MIGS to their glaucoma patients.

## Figures and Tables

**Table 1:** Summary of Principal Complications (%) by Device Type.

	iStent	iStent Inject	Hydrus	KDB	Trabectome	GATT	TRAB360	VISCO360	OMNI System	ABiC	CyPass	iStent Supra	XEN	PreserFlo	ECP
IOP spike	1.8–22.2	1.06–18.6	1.9–6.45	1.0–18.2	2.06–28.9	0–18.7	1.2	0.9–1.1	3.7	0–22.2	0.5–28.1	0	2.6–21.5	25.3–40.7	31.81
Hyphaema	1.85–11.4	0–5	1.92–6.45	0–34.9	4.72–95	0.97–38	50.6	1–13.1	3.7	1.9–20	1.3–3.1	0	0–9.6	2.5–20	0
Corneal oedema	2.1–8.97	0–10	0–3.23	1.0–15.5	0	0	6.2	0	4.9	0	0.8–3.5	0	0–2.8	1.0–17.4	18.18
Bleb needling	N/A	N/A	N/A	N/A	N/A	N/A	N/A	N/A	N/A	N/A	N/A	N/A	22.1–43.4	5.9–150	N/A
Device obstruction	0–13.2	0–6.2	0–6.2	N/A	N/A	N/A	N/A	N/A	N/A	N/A	2.1–10.2	0	3.9–8.8	0–5.7	N/A
SCH	1.8–2.27	0	0	0	1.47	0	0	1	0	0	1.6	0	0	2.5–20	0

ABiC = ab interno canaloplasty, ECP = endocyclophotocoagulation, GATT = gonioscopy-assisted transluminal trabeculotomy, IOP = intraocular pressure, KDB = Kahook Dual Blade goniotomy, SCH = subconjunctival haemorrhage.

## Data Availability

Not applicable.

## Appendix A

**Table A1:** Complication Rates of Stents Bypassing the Trabecular Meshwork.

Device	Complication	Incidence	Included Studies
iStent Trabecular Micro-Bypass Stent	IOP spike	1.8–22.2%	Ahmed et al., Al Habash et al., Donnenfeld et al., Ferguson et al., Hooshmand et al., Katz et al., Kozera et al., Nitta et al., Samuelson et al., Vold et al. [12,13,16,17,18,19,20,21,22,23,24,25]
	New cataract	1.3–20%	
	Device obstruction	0–13.2%	
	Hyphaema	1.85–11.4%	
	PCO	3–9.09%	
	Corneal oedema	2.1–8.97%	
	BCVA loss >2 lines	1.3–7.7%	
	Corneal abrasion	2.1–7.4%	
	SCH	1.8–2.27%	
	Epiretinal membrane	2%	
	Iridodialysis	0–1.85%	
	Viral keratitis	0–1.8%	
	Iris atrophy	1%	
	Blurry vision	1%	
	Dry eye	1%	
	CMO	1%	
	Foreign body sensation	0%	
	Ocular allergy	0%	
	Pain	0%	
	Stent migration	0%	
	Choroidal detachment	0%	
	Retinal detachment	0%	
	Endophthalmitis	0%	
	Hypotony maculopathy	0%	
	PAS formation	0%	
iStent Inject	IOP spike	1.06–18.6%	Al Habash et al., Berdahl et al., Fea et al., Hengerer et al., Salimi et al., Samuelson et al., Seixas et al., Shalaby et al. [28,29,30,31,32,34,35,36]
	Ocular surface disease	16.1%	
	Corneal oedema	0–10%	
	PCO	8%	
	Stent occlusion	0–6.2%	
	AC inflammation	0–6%	
	Hyphaema	0–5%	
	New cataract	4.55%	
	Ocular allergy	0–2.8%	
	Posterior vitreous detachment	2.6%	
	BCVA loss ≥2 lines	0–2.6%	
	Foreign body sensation	2.3%	
	Blurred vision	2.3%	
	Epiretinal membrane	2.3%	
	Extraocular inflammation	2.3%	
	Vitreous floaters	2.1%	
	Pain	1.06–2.1%	
	Corneal abrasion	0–2.1%	
	CMO	1.33%	
	Corneal opacity	0–1.0%	
	Hyperaemia	0.8%	
	Choroidal effusion	0%	
	Vitreous/retinal haemorrhage	0%	
	PAS formation	0%	
	Hypotony	0%	
	Endophthalmitis	0%	
	Choroidal detachment	0%	
	Myopic shift	0%	
Hydrus Microstent	PAS formation	0–18.8%	Ahmed et al., Fea et al., Lee et al., Meer et al., Pfeiffer et al. [38,39,40,41,42]
	Device obstruction	12.2%	
	BCVA loss >2 lines	0–9.68%	
	Hyphaema	1.92–6.45%	
	IOP spike	1.9–6.45%	
	Corneal oedema	0–3.23%	
	New cataract	2.6%	
	Vitreomacular traction	2.1%	
	Optic disc haemorrhage	2.0%	
	CMO	2.0%	
	Epiretinal membrane	0%	
	Stent migration	0%	
	Flat AC	0%	
	Choroidal effusion	0%	
	Hypotony	0%	
	AC inflammation	0%	
	Conjunctival injection/oedema	0%	
	Scleritis	0%	
	Corneal abrasion/infection	0%	
	Retinal tear/detachment	0%	
	Retinal haemorrhage	0%	
	Optic nerve atrophy	0%	
	Wound leak	0%	
Kahook Dual Blade Goniotomy	Hyphaema	0–34.9%	Arnljots et al., Berdahl et al., El Mallah et al., Falkenberry et al., Iwasaki et al., Lee et al., Salinas et al., Tanito et al., Ventura-Abreu et al., Wakil et al. [43,44,45,46,47,48,49,50,51,52]
	IOP spike	1.0–18.2%	
	PCO	1.6–15.5%	
	Corneal oedema	1.0–15.5%	
	CMO	3.13–6.03%	
	AC inflammation	0.54–4%	
	Descemet’s membrane tear	0–3.8%	
	Epiretinal membrane	3.45%	
	Iris prolapse	3–3.13%	
	Hypotony	0–3.13%	
	Posterior synechiae/corectopia/	2%	
	pupillary occlusion		
	Posterior vitreous detachment	1.9%	
	Retinal vein occlusion	0.18–1.72%	
	BCVA loss >2 lines	1.72%	
	Cyclodialysis cleft	1.2%	
	Vitreous haemorrhage	0.89%	
	Blood accumulation in lens bag	0.89%	
	Retinal detachment	0.86%	
	Choroidal haemorrhage	0.18%	
	Wound leak	0%	
	Flat AC	0%	
	Recurrent uveitis	0%	
	New cataract	0%	
	Endophthalmitis	0%	
	PAS formation	0%	
	Hypotony maculopathy	0%	
Trabectome	Hyphaema	4.72–95%	Ahuja et al., Avar et al., Bendel et al., Bussel et al., Esfandieri et al., Kono et al., Sato et al., Yildirim et al. [55,57,58,59,60,61,62,63]
	PAS formation	60.0%	
	IOP spike	2.06–28.9%	
	Corneal abrasion	28.5%	
	Delayed-onset hyphaema	4.9–5%	
	Iris prolapse	5%	
	BCVA loss >2 lines	0–5%	
	CMO	2.65%	
	SCH	1.47%	
	Prolonged pain	0–0.88%	
	Aqueous misdirection	0–0.4%	
	Hypotony	0–0.3%	
	Infection	0–0.3%	
	Bleb formation	0%	
	Wound leak	0%	
	Choroidal effusion	0%	
	Choroidal haemorrhage	0%	
	Flat AC	0%	
	Endophthalmitis	0%	
Gonioscopy-Assisted Transluminal Trabeculotomy	Hyphaema	0.97–38%	Baykara et al., Boese et al., Grover et al., Hamze et al., Loayza-Gamboa et al., Olgun et al., Rahmatnejad et al., Sharkawi et al. [64,65,66,67,68,70,71,72]
	IOP spike	0–18.7%	
	Fibrinous uveitis	5.4%	
	Hypotony	0–3.1%	
	Iris prolapse	2.7%	
	Iridodialysis	0–2.7%	
	Toxic anterior segment syndrome	0.93%	
	Suprachoroidal haemorrhage	0.93%	
	Descemet’s membrane detachment	0%	
	Corneal oedema	0%	
	Endophthalmitis	0%	
	Choroidal detachment	0%	
TRAB360	Hyphaema	50.6%	Sarkisian et al. [73]
	Corneal oedema	6.2%	
	Posterior vitreous detachment	2.5%	
	Iris trauma	1.2%	
	Neovascularisation	1.2%	
	Pain	1.2%	
	Steroid responder	1.2%	
	Keratic precipitates	1.2%	
	Retinal detachment	1.2%	
VISCO360	Hyphaema	1–13.1%	Heersink et al., Ondrejka et al., Tracer et al. [74,75,76]
	AC inflammation	0.9–6%	
	BCVA loss ≥2 lines	3%	
	Dry eye exacerbation	3%	
	IOP spike	0.9–1.1%	
	SCH	1%	
	Foreign body sensation	1%	
	Corneal oedema	0%	
OMNI System	PCO	17.3%	Hirsch et al. [79]
	AC inflammation	9.9%	
	CMO	4.9%	
	Corneal oedema	4.9%	
	IOP increase (≥10 mmHg above baseline >30 days post-op)	3.7%	
	Clinically significant hyphaema	3.7%	
	Worsening of VF (mean deviation ≥2 dB)	1.2%	
	BCVA loss ≥2 Snellen lines >3 months post-op	1.2%	
	Choroidal effusion	1.2%	
	Ocular allergic reaction	1.2%	
	Posterior vitreous detachment	1.2%	
	Cyclodialysis	1.2%	
Ab Interno Canaloplasty	IOP spike	0–22.2%	Al Habash et al., Davids et al., Gillmann et al., Kazerounian et al. [80,81,83,84]
	Hyphaema	1.9–20%	
	Descemet’s membrane detachment	4%	
	Iris atrophy	1.9%	
	Pupillary block	1.9%	
	AC inflammation	1.9%	
	Hypotony	0%	
	Choroidal effusion	0%	
	Choroidal haemorrhage	0%	
	Aqueous misdirection	0%	
	Wound leak	0%	

AC = anterior chamber, BCVA = best-corrected visual acuity, CMO = cystoid macular oedema, IOP = intraocular pressure, PAS = peripheral anterior synechiae, PCO = posterior capsule opacification, SCH = subconjunctival haemorrhage, VF = visual field.

**Table A2:** Complication Rates of Devices Enhancing Aqueous Outflow Through the Suprachoroidal Space.

Device	Complication	Incidence	Included Studies
CyPass Micro-Stent	IOP spike	0.5–28.1%	Gabbay et al., Grisanti et al., Hoeh et al., Reiss et al., Vold et al. [85,87,88,89,90]
	BCVA loss ≥2 lines	2.7–11.2%	
	Stent obstruction	2.1–10.2%	
	Visual field loss	1.8–10.2%	
	Iritis	8.6%	
	Choroidal effusion	4.7%	
	Corneal oedema	0.8–3.5%	
	Hyphaema	1.3–3.1%	
	Hypotony	0.4–2.9%	
	Corneal abrasion	1.9%	
	Cyclodialysis	1.9%	
	Endothelial touch	1.8%	
	Vitreous haemorrhage	1.6%	
	Myopic shift	1.6%	
	SCH	1.6%	
	Retinal detachment	0–1.6%	
	CMO	1.3–1.4%	
	Conjunctivitis	0.4–1.4%	
	Dry eye syndrome	1.3%	
	AC inflammation	0.9%	
	Stent malposition	0.9%	
	Herpes keratitis	0.4%	
	Band keratopathy	0.4%	
	Plateau iris syndrome	0.4%	
	Hypotony maculopathy	0%	
iStent Supra	BCVA loss ≥3 lines	15%	Myers et al. [94]
	Choroidal effusion	0%	
	Hyphaema	0%	
	Iridodialysis	0%	

AC = anterior chamber, BCVA = best-corrected visual acuity, CMO = cystoid macular oedema, IOP = intraocular pressure, PAS = peripheral anterior synechiae, PCO = posterior capsule opacification, SCH = subconjunctival haemorrhage.

**Table A3:** Complication Rates of Devices Shunting Aqueous Outflow into the Subconjunctival Space.

Device	Complication	Incidence	Included Studies
XEN Gel Stent	Needling required	22.1–43.4%	Baser et al., Busch et al., Grover et al., Heidinger et al., Hengerer et al., Karimi et al., Reitsamer et al., Theillac et al., Widder et al. [96,97,98,99,100,101,102,103,105]
	Hypotony	1.9–34.7%	
	Bleb fibrosis	3–24%	
	IOP spike	2.6–21.5%	
	Choroidal detachment	0–17%	
	Intraoperative bleeding	9.4%	
	Wound leak	0–9.2%	
	Stent occlusion	3.9–8.8%	
	BCVA loss ≥2 lines	0.9–6.2%	
	Hyphaema	0.9–6%	
	AC inflammation	0.9–3.1%	
	Choroidal effusion	0–3.1%	
	Endophthalmitis	0–3%	
	Corneal oedema	0–2.8%	
	Conjunctival fistula/subconjunctival cysts	2.7%	
	Iridocorneal touch	2.4%	
	Dysesthetic bleb	1.5–2.3%	
	Stent exposure	0–2.3%	
	Iris incarceration	2.1%	
	Hypotony maculopathy	1.9%	
	Stent migration	0–1.8%	
	CMO	0.5–1.7%	
	Dellen	1.5%	
	Fixed dilated pupil	1.5%	
	Macular puckering	1.5%	
	Blepharitis	1.5%	
	Chalazion	1.5%	
	Hyperaemia	1.5%	
	Shallow AC	1.3–1.5%	
	Bleb leak	0.5–1.5%	
	Aqueous misdirection	0.9–1.0%	
	Malignant glaucoma	0.9%	
	Vitreous haemorrhage	0.9%	
	Stent damage	0.4–0.9%	
	Corneal ulcer	0–0.9%	
	Dacryocystitis	0.5%	
	Macular hole	0.5%	
	Periorbital cellulitis	0.5%	
	Ptosis	0.5%	
	Scleritis	0.5%	
	Ulcerative keratitis	0.5%	
	Retinal vein occlusion	0.4–0.5%	
	Cyclodialysis cleft	0.4%	
Preserflo MicroShunt	Choroidal effusion/detachment	0–46%	Ahmed et al., Baker et al., Batlle et al., Beckers et al., Durr et al., Martinez-de-la-Casa et al., Quaranta et al., Scheres et al., Schlenker et al., Tanner et al. [106,107,108,109,110,111,112,113,114,115,116]
	IOP spike	25.3–39.5%	
	Hypotony	0–39%	
	Hyphaema/SCH	2.5–20%	
	Corneal oedema	1.0–17.4%	
	Bleb-related complications	13.0%	
	Iridocorneal touch	2.9–13.0%	
	Flat/shallow AC	0–9.4%	
	Corneal striae	8.7%	
	AC inflammation	6.8%	
	Bleb leak	0–6.6%	
	Keratitis	6.2%	
	Needling required	5.9%	
	Foreign body sensation	4.3%	
	Pain	3.3–3.7%	
	Hypotony maculopathy	0–3.5%	
	Dellen	1.2%	
	Ciliary body effusion	1.2%	
	Retinal tear	1.2%	
	Snuff out	1.2%	
	Diplopia	1.2%	
	Corneal abrasion	1.0–1.2%	
	Ptosis	0.6–1.2%	
	Vitreous haemorrhage	0–1.2%	
	CMO	0–1.2%	
	Iris incarceration	0%	
	Suprachoroidal haemorrhage	0%	
	**Late (>1–3 months)**		
	Needling required	5.0–150%	
	IOP spike	32.9–40.7%	
	Worsening visual field mean deviation	10.4%	
	Conjunctival fibrosis	8.6%	
	Device touching cornea	1.2–8.6%	
	BCVA loss ≥ 2 lines	6.1%	
	Hypotony	0–6.1%	
	Hyphaema/SCH	0–6.1%	
	Blocked/exposed shunt	0–5.7%	
	Keratitis	4.9%	
	Bleb fibrosis	4.7%	
	Iritis	2.5%	
	CMO	1.0–2.4%	
	Hypotony maculopathy	0–2.4%	
	Ptosis	0–2%	
	Corneal decompensation	0–1.8%	
	Pain	1.2%	
	Diplopia	1.2%	
	New cataract	1.2%	
	Choroidal detachment	1.2%	
	Dellen	1.2%	
	Diplopia	1.2%	
	Blebitis	0%	
	Endophthalmitis	0%	
	PAS formation	0%	
	Retinal detachment	0%	
	Flat AC	0%	

AC = anterior chamber, BCVA = best-corrected visual acuity, CMO = cystoid macular oedema, IOP = intraocular pressure, PAS = peripheral anterior synechiae, PCO = posterior capsule opacification, SCH = subconjunctival haemorrhage.

**Table A4:** Complication Rates of Device Reducing Aqueous Production by Ciliary Body Ablation.

Device	Complication	Incidence	Included Studies
ECP	IOP spike	31.81%	Izquierdo et al., Koduri et al. [119,122]
	Corneal oedema	18.18%	
	Keratitis	18.18%	
	PCO	18.18%	
	Persistent anterior uveitis	14.9%	
	CMO	3.7%	

CMO = cystoid macular oedema, ECP = endocyclophotocoagulation, IOP = intraocular pressure, PCO = posterior capsule opacification.
